# Prevalence of Phosphatidylinositol-3-Kinase (PI3K) Pathway Alterations and Co-alteration of Other Molecular Markers in Breast Cancer

**DOI:** 10.3389/fonc.2020.01475

**Published:** 2020-08-31

**Authors:** Katia Khoury, Antoinette R. Tan, Andrew Elliott, Joanne Xiu, Zoran Gatalica, Arielle L. Heeke, Claudine Isaacs, Paula R. Pohlmann, Lee S. Schwartzberg, Michael Simon, W. Michael Korn, Sandra M. Swain, Filipa Lynce

**Affiliations:** ^1^Lombardi Comprehensive Cancer Center, MedStar Georgetown University Hospital, Washington, DC, United States; ^2^Levine Cancer Institute, Charlotte, NC, United States; ^3^Caris Life Sciences, Phoenix, AZ, United States; ^4^Department of Pathology, University of Oklahoma Health Sciences Center, Oklahoma City, OK, United States; ^5^West Cancer Center, Memphis, TN, United States; ^6^Karmanos Cancer Institute, Wayne State University, Detroit, MI, United States

**Keywords:** breast cancer, molecular profiling, *PIK3CA-AKT1-PTEN* pathway, PI3K inhibitor, PD-L1, *CDH1*

## Abstract

**Background:** PI3K/AKT signaling pathway is activated in breast cancer and associated with cell survival. We explored the prevalence of PI3K pathway alterations and co-expression with other markers in breast cancer subtypes.

**Methods:** Samples of non-matched primary and metastatic breast cancer submitted to a CLIA-certified genomics laboratory were molecularly profiled to identify pathogenic or presumed pathogenic mutations in the *PIK3CA-AKT1-PTEN* pathway using next generation sequencing. Cases with loss of PTEN by IHC were also included. The frequency of co-alterations was examined, including DNA damage response pathways and markers of response to immuno-oncology agents.

**Results:** Of 4,895 tumors profiled, 3,558 (72.7%) had at least one alteration in the *PIK3CA-AKT1-PTEN* pathway: 1,472 (30.1%) harbored a *PIK3CA* mutation, 174 (3.6%) an *AKT1* mutation, 2,682 (54.8%) had PTEN alterations (*PTEN* mutation in 7.0% and/or PTEN loss by IHC in 51.4% of cases), 81 (1.7%) harbored a *PIK3R1* mutation, and 4 (0.08%) a *PIK3R2* mutation. Most of the cohort consisted of metastatic sites (*n* = 2974, 60.8%), with *PIK3CA* mutation frequency increased in metastatic (32.1%) compared to primary sites (26.9%), *p* < 0.001. Other *PIK3CA* mutations were identified in 388 (7.9%) specimens, classified as “off-label,” as they were not included in the FDA-approved companion test for *PIK3CA* mutations. Notable co-alterations included increased PD-L1 expression and high tumor mutational burden in *PIK3CA-AKT1-PTEN* mutated cohorts. Novel concurrent mutations were identified including *CDH1* mutations.

**Conclusions:** Findings from this cohort support further exploration of the clinical benefit of PI3K inhibitors for “off-label” *PIK3CA* mutations and combination strategies with potential clinical benefit for patients with breast cancer.

## Introduction

Phosphatidylinositol-3-kinase/protein kinase B (PI3K/AKT) signaling pathway is dysregulated in various carcinomas including breast cancer, through several genomic abnormalities. Gain-of-function mutations of PI3K subunits including *PIK3CA* encoding the catalytic subunit p110α, *PIK3CB* encoding subunit p110β, and genes like *PIK3R1* encoding regulatory subunit p85α have been described. *PIK3CA* involves two main “hotspots” for activating mutations: E545K of the helical domain on exon 9, and H1047R of the kinase domain on exon 20 (1–9). Mutations in *AKT1*, and loss-of-function mutations and alterations in *PTEN* also affect the PI3K pathway, thus promoting cell survival and resistance (8, 10, 11).

About 40% of HR-positive breast cancers harbor *PIK3CA* mutations. Alpelisib (PIQRAY, Novartis Pharmaceuticals Corporation), a PI3K inhibitor, received FDA approval in combination with fulvestrant for patients with hormone receptor (HR)-positive, human epidermal growth factor receptor 2 (HER2)-negative *PIK3CA*-mutated advanced breast cancer. Approval was based on SOLAR-1, a phase 3 randomized trial that showed a benefit of 5.3 months in progression-free survival with the addition of alpelisib in the cohort of patients with *PIK3CA*-mutated breast cancer (12). *PIK3CA* mutations that were considered for trial enrollment in SOLAR-1 included C420R, E542K, E545A, E545D (1635G > T only), E545G, E545K, Q546E, Q546R, H1047L, H1047R, and H1047Y. The FDA also approved the therascreen^®^ *PIK3CA* RGQ PCR Kit, (QIAGEN Manchester, Ltd.), a companion test able to select patients who have these specific mutations. For the purpose of the current report, mutations detectable by the companion test were considered alpelisib “on-label” (12).

The use of PI3K and AKT inhibitors in combination with chemotherapy, endocrine therapy, HER2-targeted therapies, cyclin-dependent kinase 4/6 (CDK4/6) and poly ADP ribose polymerase (PARP) inhibitors, has been explored in pre-clinical and clinical models across multiple tumor types (13–27). Based on emerging data that loss of PTEN may represent a potential mechanism for resistance to checkpoint inhibitors, combinations of a triplet regimen of AKT inhibitor, programmed death-ligand 1 (PD-L1) inhibitor and chemotherapy are also being explored in the setting of advanced breast cancer (28).

In this study, we report the prevalence of PI3K pathway alterations and co-expression with other markers of clinical interest in different breast cancer subtypes, based on somatic molecular profiling. This approach can lead to the identification of novel drug combinations with potential synergy that could be further evaluated in the clinical trial setting.

## Methods

### Study Design

A retrospective review of molecular profiles was performed for 4,845 female and 50 male breast cancer cases submitted to Caris Life Sciences, a Clinical Laboratory Improvement Amendments (CLIA)/College of American Pathologists (CAP)/ISO15189/New York State Department of Health (NYSDOH)-certified clinical laboratory (Phoenix, AZ), between January 2015 and June 2019. Specimens were obtained from more than 500 centers, primarily within the United States, and patient demographics were de-identified (29–31).

### Immunohistochemistry (IHC)

IHC was performed on formalin-fixed paraffin-embedded (FFPE) sections of glass slides. Slides were stained using automated staining techniques (Benchmark XT, Ventana, Tucson, AZ; and Autostainer Link 48, Dako, Carpenteria, CA), per the manufacturer's instructions, and were optimized and validated per CLIA/CAO and International Organization for Standardization (ISO) requirements. Staining was scored for intensity (0 = no staining; 1+ = weak staining; 2+ = moderate staining; 3+ = strong staining) and cancer cell staining percentage (0–100%). Results were categorized as positive or negative by defined thresholds specific to each marker based on published clinical literature. The cutoff for ER and PR positivity was ≥1% staining according to the American Society of Clinical Oncology/College of American Pathologists (ASCO/CAP) guidelines (29–31) (Table S1). A board-certified pathologist evaluated all IHC results independently. For PD-L1, a laboratory developed test (LDT) Ventana SP142 assay was initially used to test for PD-L1 expression of tumor cells, and after FDA approval in March 2019, the FDA CDx Ventana PD-L1 (SP142) assay was used to test for PD-L1 expression of immune cells.

### Chromogenic *in situ* Hybridization (CISH)

*HER2/neu* amplification was examined by CISH (INFORM HER2 Dual ISH DNA Probe Cocktail, Ventana, Tucson, AZ). All HER2 test results were classified according to the 2018 ASCO/CAP HER2 testing recommendations (31, 32).

### Next-Generation Sequencing (NGS)

NGS was performed on genomic DNA isolated from FFPE tumor samples using the NextSeq platform (Illumina, Inc., San Diego, CA). Matched normal tissue was not sequenced. A custom-designed SureSelect XT assay was used to enrich 592 whole-gene targets (Agilent Technologies, Santa Clara, CA). All variants were detected with > 99% confidence based on allele frequency and amplicon coverage, with an average sequencing depth of coverage of >500 and an analytic sensitivity of 5%. Prior to molecular testing, tumor enrichment was achieved by harvesting targeted tissue using manual microdissection techniques. Genetic variants identified were interpreted by board-certified molecular geneticists and categorized as “pathogenic,” “presumed pathogenic,” “variant of unknown significance (VUS),” “presumed benign,” or “benign,” according to the American College of Medical Genetics and Genomics (ACMG) standards. When assessing mutation frequencies of individual genes, “pathogenic,” and “presumed pathogenic” were counted as mutations while “benign,” “presumed benign” variants, and “VUS” were excluded.

### Microsatellite Instability (MSI)/Mismatch Repair (MMR) Status

Up to three different testing methods were used to determine MSI/MMR status of tumors profiled, including Fragment Analysis (FA), IHC, and NGS. FA was tested with Microsatellite Instability Analysis (Promega, Madison, WI), which included fluorescently labeled primers for co-amplification of seven markers including five mononucleotide repeat markers (BAT-25, BAT26, NR-21, NR24, and MONO-27) and two pentanucleotide repeat markers (Penta C and D). The mononucleotide markers were used for MSI determination, while the pentanucleotide markers were used to detect either sample mix-ups or contamination. A tumor sample was considered MSI if two or more mononucleotide repeats were abnormal; if one mononucleotide repeat was abnormal or repeats were identical between the tumor and adjacent normal tissue, then the tumor sample was considered microsatellite stable (MSS). MMR protein expression was tested by IHC (using the following antibody clones: MLH1, M1 antibody; MSH2, G2191129 antibody; MSH6, 44 antibody, and PMS2, EPR3947 antibody [Ventana Medical Systems, Inc., Tucson, AZ, USA]). The complete absence of protein expression of any of the four MMR proteins tested (0 intensity in 100% of cells) was considered MMR deficient (dMMR). NGS method for measuring MSI (MSI-NGS) used over 7,000 target microsatellite loci and compared to the reference genome hg19 from the University of California, Santa Cruz (UCSC) Genome Browser database. The number of microsatellite loci that were altered by somatic insertion or deletion was counted for each sample. Only insertions or deletions that increased or decreased the number of repeats were considered. Genomic variants in the microsatellite loci were detected using the same depth and frequency criteria as used for mutation detection. MSI-NGS results were compared with results from over 2,000 matching clinical cases analyzed with traditional PCR-based methods. The threshold to determine MSI by NGS was determined to be 46 or more loci with insertions or deletions to generate a sensitivity of >95% and specificity of >99%. The three platforms generated highly concordant results as previously reported, and in the rare cases of discordant results, the MSI or MMR status of the tumor was determined in the order of FA, IHC, and NGS (33).

### Tumor Mutational Burden (TMB)

TMB was measured (592 genes and 1.4 megabases [MB] sequenced per tumor) by counting all non-synonymous missense mutations found per tumor that had not been previously described as germline alterations. The threshold to define TMB-high was ≥10 mutations/MB (34).

### Statistical Analysis

The proportion of pathogenic or presumed pathogenic co-alterations (mutation and/or expression) identified from all tumor specimens tested for each specific mutation were calculated and compared between mutated (MT) and wild type (WT) breast tumors, defined based on the presence of *PIK3CA-AKT-PTEN* alterations, and among the breast cancer subtypes. Sequencing tests with indeterminate results due to low depth of coverage were excluded from the total number for percentage calculation. The total frequency of *PIK3CA-AKT-PTEN*-MT cases in the complete cohort and per subtype was calculated by dividing the number of tumors with at least one alteration in *PIK3CA, AKT1*, or *PTEN* by the total number of tumors tested. Statistical analysis was performed using Chi-square tests. *P* < 0.05 were considered statistically significant. The log2 odds ratio was calculated for biomarker pairs to assess the tendency of mutual exclusivity (value ≤ 0) or co-occurrence (value > 0), with *p*-values derived from a one-sided Fisher's exact test and *q*-values derived from a Benjamini-Hochberg correction procedure to decrease the false discovery rate.

## Results

### Description of Breast Cancer Cases

A total of 4,895 breast cancer cases from 2015 to 2019 with available *PIK3CA, AKT1*, and *PTEN* NGS results were reviewed. Four thousand eight hundred and forty-five (99%) were female, and fifty (1%) were male. The median age at the time of testing was 58 years (17–90, *SD* = 12.8). One thousand nine hundred and twenty-one (39.2%) tissue samples were from primary sites, and 2,974 (60.8%) were from metastatic sites. Metastatic samples included 776 (26.1%) from liver, 320 (10.8%) from bone, 319 (10.7%) from lung, and 135 (4.5%) from brain. Two hundred and fifty cases (5.1%) were lobular. Two thousand five hundred and forty-nine (52.1%) cases were HR-positive HER2-negative, 1,863 (38.1%) were triple negative breast cancer (TNBC), 263 (5.4%) were HR-positive, HER2-positive, and 220 (4.5%) were HR-negative, HER2-positive (Table 1). One thousand eight hundred and fifty-five of 1863 TNBC samples (99.6%) had IHC Androgen Receptor (AR) results available, with 376 (20.3%) samples positive for AR expression.

**Table 1:** Patient and tumor characteristics.

**Variable**	**All cases**	**HR + HER2-**	**TNBC**	**HR + HER2+**	**HR - HER2+**
All cases, *N* (%)	4,895 (100)	2,549 (52.1)	1,863 (38.1)	263 (5.4)	220 (4.5)
Female cases, *N* (%)	4,845 (99)	2,515 (98.7)	1,851 (99.4)	261 (99.2)	218 (99.1)
Male cases, *N* (%)	50 (1)	34 (1.3)	12 (0.6)	2 (0.8)	2 (0.9)
Median age, years (*SD*)	58 (12.8)	59 (12.5)	57 (13.1)	55 (12.9)	55 (12.3)
Age range (year)	17–90	17–90	19–90	19–90	26–90
Primary site, *N* (%)	1,921 (39.2)	889 (34.9)	831 (44.6)	111 (42.2)	90 (40.9)
Metastatic site, *N* (%)	2,974 (60.8)	1,660 (65.1)	1,032 (55.4)	152 (57.8)	130 (59.1)

### Prevalence of *PIK3CA, AKT1*, and *PTEN* Mutations

Three thousand five hundred and fifty-eight (72.7%) cases had at least one alteration in the *PIK3CA-AKT1-PTEN* pathway: 1,472 (30.1%) of cases harbored a *PIK3CA* mutation, 174 (3.6%) harbored an *AKT1* mutation, 2,682 (54.8%) had *PTEN* alterations including 344 (7.0%) *PTEN* mutations, and/or PTEN loss by IHC in 2,516 (51.4%) cases. Eighty-one (1.7%) tumors harbored a *PIK3R1* mutation, with 66 unique alterations identified (including 4 pathogenic, 55 presumed pathogenic), and 4 (0.08%) cases harbored a *PIK3R2* mutation (G373R mutation). The most common hotspot mutations in *PIK3CA* were in the kinase domain (H1047R in 567 (38.5%) of all *PIK3CA* alterations) and in the helical domain (E545K in 304 (20.7%) of all *PIK3CA* alterations) (4–8, 35). Importantly, alpelisib “off-label” activating *PIK3CA* mutations (those not detected by therascreen^®^ *PIK3CA* RGQ PCR Kit in SOLAR-1 trial) were seen in 388 (7.9%) of all breast tumors (12). The most common off-label mutations included: N345K (*n* = 74), E726K (*n* = 45), G1049R (*n* = 19), and Q546K (*n* = 15) (35, 36). The most common *AKT1* mutation was in the single hotspot mutation E17K in 164 (94.3%) of all *AKT1* mutations. Table S2 describes the most common *PIK3CA-AKT1-PTEN* pathway-associated genes and their frequencies in our cohort.

**Figure 1:**
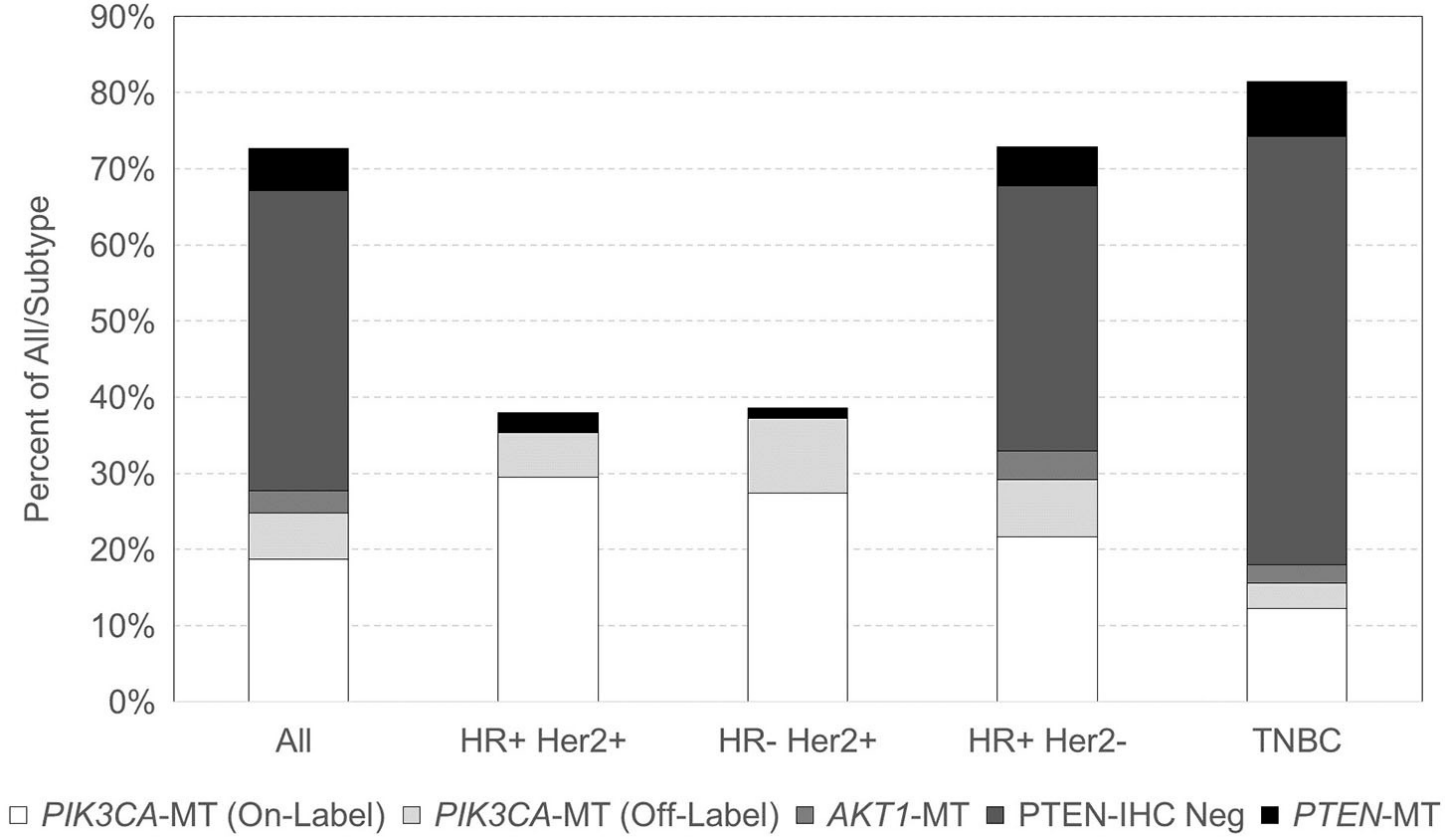
Distribution of *PIK3CA, AKT1*, and *PTEN* mutations across breast cancer subtypes.

With respect to breast cancer subtypes, 100/263 (38.0%) HR-positive HER2-positive, 85/220 (38.6%) HR-negative HER2-positive, 1,857/2,549 (72.9%) HR-positive HER2-negative, and 1,516/18,63 (81.4%) TNBC samples had at least one alteration in the *PIK3CA-AKT1-PTEN* pathway (Figure 1). Among TNBC samples with a *PIK3CA-AKT1-PTEN* pathway alteration, 1,192 (78.6%) were negative for AR expression by IHC (i.e., quadruple-negative breast cancer). *PIK3CA* was the most frequent alteration in HER2-positive breast cancer, in 178/185 (96.2%) cases. Within HER2-negative subtypes, *PTEN* was the most frequent alteration with *PTEN* mutation or PTEN loss by IHC present in 79.3% of mutated cases. TNBC was the subtype with the lowest frequency of *PIK3CA* mutations (18.0% in TNBC vs. 37% in other subtypes). No *AKT1* mutations were noted in HER2-positive tumors, and they were found in low frequencies in HER2-negative tumors (6.2%). *PIK3CA* mutation frequency increased with age, with mutations identified in 26.1% of tumors from patients younger than 60 years and in 35.0% of patients 60 years or older (*p* < 0.0001). Specifically, in HR-positive HER2-negative, *PIK3CA* mutations were found in 42.0% of tumors in age ≥60 vs. 33.5% in age <60 (*p* < 0.0001), and in TNBC at 23.5% in age ≥60 vs. 14.1% in age <60 (*p* < 0.0001). *PIK3CA* mutations were increased overall in metastatic vs. primary sites (32.1 vs. 26.9%, *p* < 0.0001), specifically in HR-positive HER2-positive breast cancer (44.1 vs. 25.2%, respectively, *p* = 0.002) and TNBC (21 vs. 14.3%, respectively, *p* = 0.0002). Table S3 also describes the patient and tumor characteristics by *PIK3CA, AKT1*, and *PTEN* mutation status.

### Co-alterations in *PIK3CA, AKT1*, and *PTEN* Individually Mutated and Co-mutated Cohorts

Figure S1 illustrates the prevalence and co-occurrence of *PIK3CA, AKT1*, and *PTEN* alterations in breast tumors. Table 2 summarizes the biomarker subgroup co-alterations for all subtypes. Of the *PIK3CA-AKT1-PTEN*-MT cases (*n* = 3,558), only 5 (0.1%) cases had co-occurring alterations in *PIK3CA, AKT1*, and *PTEN*.

**Table 2:** Summary of biomarker subgroup co-alterations for all subtypes.

**Pathway**	**Gene/protein**	***PIK3CA-AKT1-***	***PIK3CA***	***AKT1***	***PTEN***	***PIK3CA-PTEN***	***AKT1-PTEN***	***PIK3CA-AKT1-***
		***PTEN* WT (%)**	**MT (%)**	**MT (%)**	**MT (%)**	**MT (%)**	**MT (%)**	*PTEN* MT^*^ (%)
Homologous recombination	*BRCA1*	2.7	0.5^*^	0.0	5.3^**^	0.3^*^	0.0	3.0
	*BRCA2*	5.4	3.9	0.0	5.2	2.3^*^	1.1	4.1
	*PALB2*	1.3	0.3^*^	1.4	0.8	0.8	1.1	0.7^*^
Possible predictors of IO benefit	PD-1	50.0	43.4	66.7	57.3	34.0	66.7	49.7
	PD-L1 TC (SP142)	4.2	3.2	1.6	8.7^**^	6.8^**^	5.7	6.9^**^
	PD-L1 IC (SP142)	26.6	16.2	25.0	39.7^**^	13.3^*^	25.0	29.2
	MSI	0.6	0.3	0.0	0.9	1.2	0.0	0.7
	TMB-High (≥10/Mb)	18.8	27.1^**^	24.3	19.8	26.7^**^	21.3	22.9^**^
Chromatin remodeling	*ARID1A*	18.6	18.6	5.6	9.1^*^	16.0	2.8^*^	12.4^*^
	*ARID2*	0.8	0.8	1.5	0.4	1.2	0.0	0.7
*RAS-RAF-MEK-ERK*	*HRAS*	0.1	0.8^**^	0.0	0.4	1.5^**^	1.1	0.7^**^
	*KRAS*	1.1	2.1	1.4	1.5	3.2^**^	1.1	2.0^**^
	*NRAS*	0.0	0.3	0.0	0.2	0.3^**^	0.0	0.2
	*BRAF*	0.1	0.8^**^	0.0	0.3	0.5	5.4^**^	0.5
Others	*TP53*	53.1	47.0^*^	43.9	72.1^**^	46.7^*^	56.2	60.9^**^
	*CDH1*	6.1	15.8^**^	20.3^**^	4.8	18.4^**^	10.6	10.3^**^
	*NF1*	2.1	5.6^**^	12.1^**^	4.4^**^	11.7^**^	7.2^**^	6.2^**^
	*RB1*	2.6	3.8	3.1	6.2^**^	6.0^**^	4.3	5.5^**^
	*ERBB2*	3.4	3.9	1.4	1.7	2.3	0.0	2.3^*^

PIK3CA-AKT1-PTEN-MT^*^ = pathogenic alteration in PIK3CA, AKT1, or PTEN.

*All other MT cohorts = pathogenic alteration in genes included in cohort name and no mutation in genes not included in cohort name for PIK3CA, AKT1, and PTEN*.

*WT = no pathogenic mutations in PIK3CA, AKT1, and PTEN*.

*TMB = tumor mutational burden*.

* /

** *= statistically significant decrease/increase, respectively between MT and WT cohorts*.

### Co-mutation Frequency With *PIK3CA-AKT1-PTEN* Alteration

The co-existence of *PIK3CA-AKT1-PTEN* alterations with other alterations in pathways of clinical relevance was explored, including genes involved in homologous recombination (HR) and DNA damage sensors, chromatin remodeling, RAS-RAF-MEK-ERK pathway, and potential predictors of benefit to immunotherapy. The frequency of selected co-mutations with *PIK3CA-AKT1-PTEN* alterations is illustrated in Table 3. There was overall low co-alteration frequency for the HR deficiency (HRD)-related genes across all subtypes. *CDH1, NF1*, and *RB1* mutations were significantly increased in the *PIK3CA-AKT1-PTEN*-MT cohort, particularly in HR-positive and TNBC subtypes.

**Table 3:** Selected co-alterations based on statistical significance.

**Pathway**	**Gene/ protein**	**All subtypes**		**HR+** **HER2+**		**HR- HER2+**		**HR+HER2-**		**TNBC**	
		**(%)**		**(%)**		**(%)**		**(%)**		**(%)**	
		**MT**	**WT**	**MT**	**WT**	**MT**	**WT**	**MT**	**WT**	**MT**	**WT**
Homologous recombination	*BRCA1*	3.0	2.7	1.0	0.6	0.0	0.8	0.8^*^	1.8^*^	6.1	6.0
	*BRCA2*	4.1	5.4	1.0	2.5	3.6	2.3	4.1^*^	7.9^*^	4.4	3.0
	*PALB2*	0.7^*^	1.3^*^	0.0	0.0	0.0	0.0	0.5^*^	2.5^*^	0.9	0.3
DNA Damage Sensors	*ATM*	1.6	2.3	2.0	3.1	1.2	1.5	1.9	2.9	1.2	1.2
	*ATRX*	1.0	0.6	0.0	0.0	2.3	1.4	1.2	0.5	0.7	0.6
	*CHEK2*	2.0	1.5	3.6	1.4	1.3	1.6	2.9	2.1	1.0	0.3
Possible predictors of CPI benefit	PD-1	49.8	50.0	38.5	52.2	66.7	66.7	37.5	41.7	66.0	58.1
	PD-L1 TC (SP142)	6.9^*^	4.2^*^	3.3	0.7	8.1	4.9	3.3	3.0	11.6	8.1
	PD-L1 IC (SP142)	29.2	26.6	50.0	18.8	25.0	27.3	12.9	16.7	42.1	40.4
	MSI	0.7	0.6	0.0	0.0	1.2	0.8	0.8	0.4	0.7	1.2
	TMB-High (≥10/Mb)	22.9^*^	18.8^*^	36.0^*^	17.2^*^	29.8	25.9	21.2^*^	16.6^*^	23.6	21.3
Chromatin remodeling	*ARID1A*	12.4^*^	18.6^*^	19.4	23.8	7.1	18.9	17.1	21.5	5.8	9.6
	*ARID2*	0.7	0.8	0.0	1.9	0.0	0.7	0.8	0.6	0.6	0.9
*RAS-RAF-MEK-ERK*	*HRAS*	0.7^*^	0.1^*^	0.0	0.0	0.0	0.0	0.2	0.1	1.4	0.3
	*KRAS*	2.0^*^	1.1^*^	1.0	0.0	0.0	0.0	1.9	1.2	2.2	2.0
	*NRAS*	0.2	0.0	0.0	0.0	0.0	0.0	0.2	0.0	0.3	0.0
	*BRAF*	0.5	0.1	1.0	0.0	1.2	0.0	0.6	0.3	0.4	0.0
Others	*TP53*	60.9^*^	53.1^*^	66.3	60.8	85.5	86.7	38.4^*^	25.6^*^	84.7	86.4
	*CDH1*	10.3^*^	6.1^*^	9.1^*^	2.5^*^	4.8	2.2	15.1^*^	9.6^*^	4.9^*^	2.3^*^
	*NF1*	6.2^*^	2.1^*^	9.5^*^	0.8^*^	2.7	3.6	5.8^*^	1.8^*^	6.7^*^	2.8^*^
	*RB1*	5.5^*^	2.6^*^	2.2	1.3	3.6	3.0	3.9^*^	1.7^*^	7.8	5.0
	*ERBB2*	2.3^*^	3.4^*^	3.0	3.1	3.5	6.7	3.2	3.5	1.0^*^	2.3^*^

*MT = at least 1 pathogenic mutation in PIK3CA, AKT1, or PTEN or PTEN loss by IHC; WT = no pathogenic mutations in PIK3CA, AKT1, and PTEN; TMB = tumor mutational burden; TC, tumor cells; IC, immune cells; CPI, immune checkpoint inhibitors*.

* *Statistically significant difference between MT and WT*.

**Figure 2:**
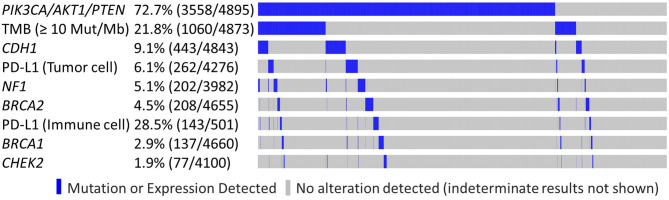
Genomic features observed in *PIK3CA/AKT1/PTEN* altered and non-altered tumors. Oncoprint plot illustrates co-occurrence of possible driver events and events associated with mutual exclusivity.

LDT PD-L1 tumor cells (TC) antibody was used in 4,276/4,895 samples (87.4%), 501/4,895 (10.2%) were tested with FDA CDx PD-L1 immune cell (IC) stain and 21/4,985 (0.4%) were tested with both methods. This difference reflects the change in practice after the approval of the FDA CDx Ventana PD-L1 (SP142) assay. TMB-high and LDT PD-L1 (TC) expression frequency was increased in the *PIK3CA-AKT1-PTEN*-MT cohort (*p* < 0.05), whereas the frequency of FDA CDx PD-L1 (IC) expression was not significantly different between MT and WT cohorts (Table 3). In the *RAS* signaling pathway, there was an increased *HRAS, KRAS*, and *NRAS* co-mutation frequency in the MT cohort across all subtypes, with no *HRAS* or *NRAS* mutations identified in the HER2-positive subtypes, and no *KRAS* mutations identified in the HR-negative HER2-positive subtype. *BRAF* co-mutation frequency was increased in *PIK3CA-AKT1-PTEN*-MT cohort across all subtypes (p ns); however, *BRAF* co-mutation frequency was very low for both MT and WT cohorts. Other statistically significant increased co-alterations between *PIK3CA-AKT1-PTEN*-WT and *PIK3CA-AKT1-PTEN*-MT cohorts were seen with *TP53* (53.1 vs. 60.9%), *CDH1* (6.1 vs. 10.3%), *NF1* (2.1 vs. 6.2%), and *RB1* (2.6 vs. 5.5%). The frequency of *CDH1* mutations in *PIK3CA-AKT1-PTEN*-MT was higher in lobular than in non-lobular carcinoma (73.5 vs. 6.8%), although frequency of *CDH1* mutations remained positively associated with *PIK3CA-AKT-PTEN*-MT after lobular cases were excluded from the analysis. We evaluated the co-occurrence of possible driver events and events associated with mutual exclusivity using an Oncoprint plot (Figure 2). Genomic features were selected based on mutual exclusivity analysis that identified TMB, *CDH1, NF1*, PD-L1, *CHEK2*, and *BRCA1/2* as significant tendencies of co-occurrence.

### Alpelisib On/Off-Label *PIK3CA* Mutations in Breast Cancer

**Figure 3:**
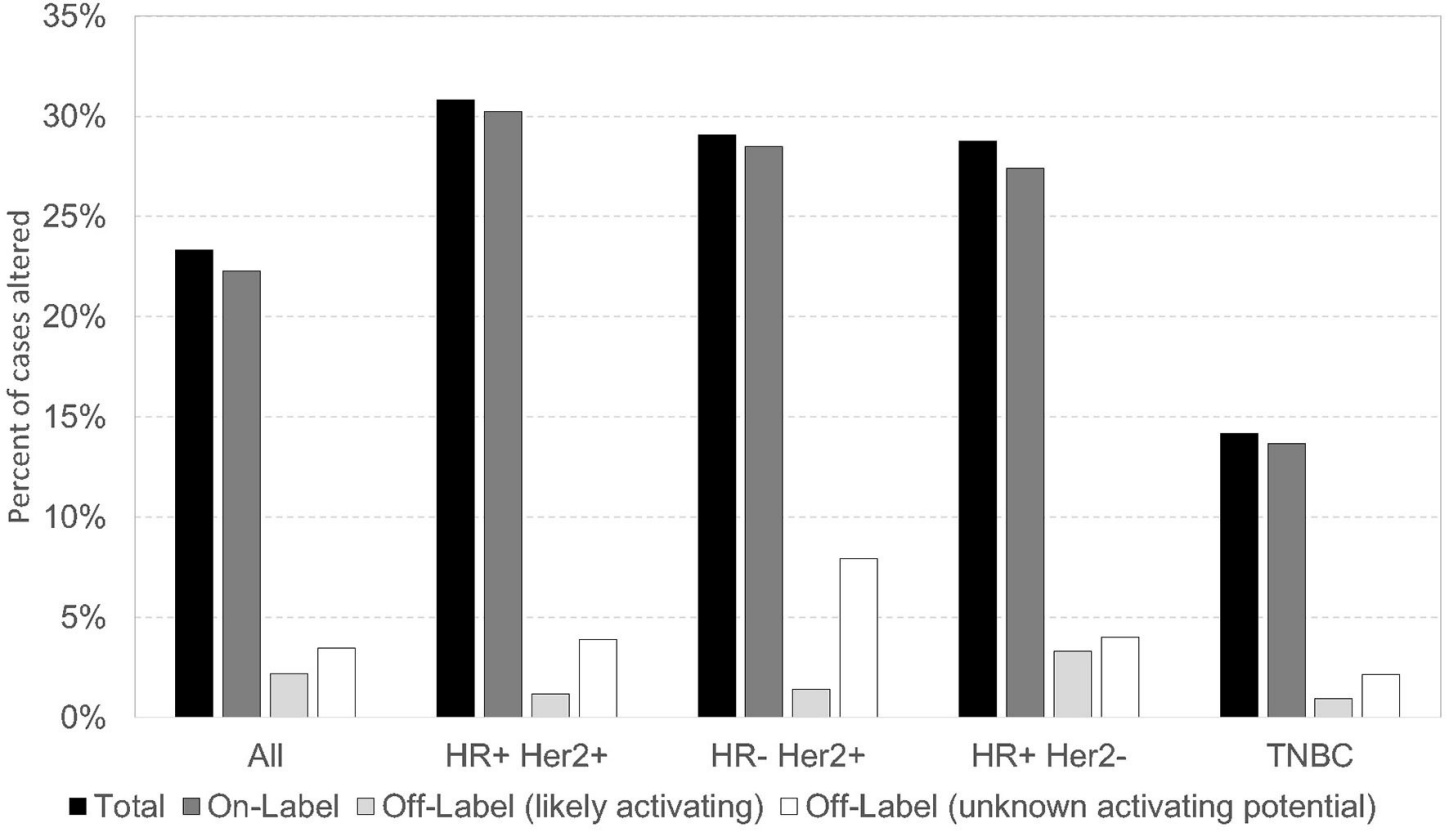
Prevalence of alpelisib On/Off Label *PIK3CA* mutations across breast cancer subtypes.

In this cohort, *PIK3CA* pathogenic/presumed pathogenic mutations (*n* = 1616) were classified as on-label (if included in the SOLAR-1 trial) or off-label. There were 1,204 (74.5%) *PIK3CA* on-label mutations, and 412 (25.5%) *PIK3CA* off-label mutations. Of all *PIK3CA* mutations identified, 57/1,616 (3.5%) were off-label mutations (56 pathogenic/presumed pathogenic, 1 VUS) at amino acid positions that correspond to those of on-label mutations. Some of these off-label mutations have been described as activating in preclinical studies, including N345K, Q546K, and G1049R. In our study, N345K (*n* = 74), Q546K (*n* = 15), and G1049R (*n* = 19) comprised 108/412 (~26%) of the off-label pathogenic/presumed pathogenic mutations (35, 36). Other novel off-label pathogenic/presumed pathogenic mutations have not been defined as activating vs. deleterious. The prevalence of alpelisib on/off-label *PIK3CA* mutations was similar across breast cancer subtypes, with the lowest frequency seen in TNBC. More than 60% of alpelisib on/off-label mutations occurred in HR-positive HER2-negative subtype (Figure 3). The alpelisib on-label (*n* = 1,040) and off-label (*n* = 264) cohorts included cases with exclusively on- or off-label mutations, respectively. Cases with *PIK3CA* VUS mutations (*n* =1 14) and cases with both alpelisib on- and off-label mutations in the same tumor (*n* = 115) were excluded from the analysis.

Few co-alterations were significantly different between alpelisib on-label and off-label *PIK3CA*-mutant cohorts, illustrated in Table S4. In all breast cancer subtypes, there was an increased co-mutation frequency in alpelisib off-label cohort compared to on-label cohort in *CHEK2* and *ERBB2*, and increased co-mutation frequency in alpelisib on- and off-label cohorts compared to *PIK3CA*-WT in *CHEK2, HRAS, TP53, CDH1*, and *NF1*.

## Discussion

In this large cohort of 4,895 NGS molecularly profiled breast tumors, we observed a high prevalence of mutations in the *PIK3CA-AKT1-PTEN* pathway, in up to 72.7% of tumors across all breast cancer subtypes. Other studies have described distinct prevalence of mutations in the *PIK3CA-AKT-PTEN* pathway, varying from 38.9% *PIK3CA* mutations in the METABRIC database, 25% pathway mutations in the PAKT trial and 41% pathway mutations in the LOTUS trial (37–39). This variation likely reflects different criteria defining an altered pathway, distinct assays and variant calling or differences in patient populations. The majority of our tumor samples were from metastatic sites (60.8%), while the entire METABRIC database and 82% of the PAKT samples were obtained from primary sites (38, 39). The PAKT and the LOTUS trials enrolled patients with TNBC only (37, 38). In addition, there may also be racial and ethnic differences in the prevalence of *PIK3CA-AKT1-PTEN* alterations. It has been previously reported that there were differences on the location of *PIK3CA* mutations and were overall less prevalent in African Americans compared to Caucasians (40). In the LOTUS trial, nearly half of the patients were of Asian ethnicity (37).

As previously reported, *PIK3CA* was commonly mutated in HR-positive subtypes (37.6%), in a higher percentage of cases than in SOLAR-1 (29%) which can be explained by a broader number of *PIK3CA* alterations included in our analysis (12, 41). *PIK3CA* was also the most frequent alteration in HER2-positive breast cancer. *PTEN* alterations mostly occurred in HER2-negative subtypes and were present in more than half of tumors tested (54.8%), by mutation and/or *PTEN* loss by IHC. *AKT1* mutations were rare, with none identified in HER2-positive tumors. This type of information may be relevant for clinical trial design. Recent phase II and III trials using AKT inhibitors have not used a specific biomarker selected population for trial participation, however, trials conducted with alpelisib enrolled a biomarker selected population (38, 42).

The use of immunotherapy in combination with chemotherapy has been established as the new standard of care in advanced PD-L1 positive TNBC with improved outcomes seen in IMpassion 130 trial (43). In our cohort, the most notable co-alteration identified was a significant increase in PD-L1 expression in tumor cells and high TMB in *PIK3CA-AKT1-PTEN* mutated cohorts, especially in HR-positive subtypes. This finding could form a basis for further development of drug combinations that affect the *PIK3CA-AKT1-PTEN* pathway in combination with agents that target the immune system. Such studies are underway, and include for example, a Phase Ib trial evaluating the safety and efficacy for ipatasertib, an AKT inhibitor, combined with atezolizumab and paclitaxel or nab-paclitaxel in patients with advanced TNBC, which showed an objective response rate of 73% for the combination in 26 patients at a median follow up of 6.1 months, regardless of PD-L1 or *PIK3CA-AKT1-PTEN* status (28). A phase III trial is currently underway for patients with advanced TNBC, evaluating the use of paclitaxel with ipatasertib vs. placebo, and atezolizumab vs. placebo for non-PD-L1 positive patients, and paclitaxel with atezolizumab and ipatasertib vs. placebo for PD-L1 positive patients (44).

Of interest, most cases of *CDH1* mutations also demonstrated concurrent mutations in the *PIK3CA-AKT1-PTEN* pathway and/or high TMB, regardless of histology. Of the 443 total *CDH1*-MT cases included in the cohort, none had a *ROS1* mutation or fusion, indicating that *CDH1* and *ROS1* result in synthetic lethality, as has been previously described in breast cancer (45). *In vivo*, inhibition of *ROS1* has been shown to produce significant antitumor effects in different models of E-cadherin-defective breast cancer. Therefore, *ROS1* inhibitors may be of benefit for patients with *CDH1* mutated breast cancers, in combination with PI3K or AKT inhibitors, with or without immunotherapy, and warrant further investigation in early clinical trials.

In this report, the most common hotspot mutations in *PIK3CA* were in the kinase domain and helical domain, considered on-label mutations. However, off-label activating *PIK3CA* mutations were also seen, and we identified pathogenic and presumed pathogenic mutations not previously defined, with more than half (293/412, 71.1%) of cases occurring in HR-positive, HER2-negative subtype. At this point, not all novel off-label mutations have data regarding their functional consequences (i.e., activating vs. deleterious), and only a few off-label mutations have been previously reported as deleterious in preclinical studies (35, 36). Therefore, it remains difficult to interpret the functional consequences of new genetic mutations, and the efficacy of PI3K inhibitors in tumors with alpelisib off-label mutations remains unknown. Notable co-alterations seen in off-label mutations include *CHEK2* mutations and MSI-High/TMB-High, which may lead to novel targets and drug combinations, and wider use of NGS molecular profiling, given that the current FDA-approved companion test includes only on-label mutations tested in SOLAR-1 (12).

The major limitations of this study are the lack of matched clinical data and outcomes, and therefore the clinical implications of our findings remain to be determined. In addition, the use of molecular profiling in this dataset was determined by clinicians and may have been influenced by patient and tumor characteristics. Therefore, the actual incidence of alterations in the *PIK3CA-AKT1-PTEN* pathway here described may not fully represent the general population. Lastly, given the lack of known activating potential of most “off-label” *PIK3CA* mutations, the clinical implications of our findings remain to be determined.

In conclusion, we showed that the prevalence of alterations in the *PIK3CA-AKT1-PTEN* pathway is elevated across all tumor subtypes and that a considerable number of tumors harbor off-label mutations. This is a very large and comprehensive dataset that characterized the *PIK3CA-AKT1-PTEN* pathway beyond *PIK3CA* mutations and included both primary and metastatic breast cancer cases. Although our dataset lacks outcome data, we believe that our results are hypothesis-generating and it is worth exploring the clinical implications of the “off-label” *PIK3CA* mutations. Finally, our data identified potential targets of interest for combination strategies and support the continuous investigation of the use of agents targeting the *PIK3CA-AKT1-PTEN* pathway in combination with immunotherapy.

## Data Availability Statement

The datasets presented in this article are not readily available because the raw data is protected proprietary information. Requests to access the datasets should be directed to aelliott@carisls.com of Caris Life Sciences.

## Ethics Statement

Ethical review and approval was not required for the study on human participants in accordance with the local legislation and institutional requirements. Written informed consent from the patients was not required to participate in this study in accordance with the national legislation and the institutional requirements.

## Author Contributions

KK, AT, AE, JX, ZG, AH, CI, PP, LS, MS, WK, SS, and FL contributed to the design, implementation of the research, to the analysis of the results, and to the writing of the manuscript. All authors contributed to the article and approved the submitted version.

## Conflict of Interest

AT reports consulting/advisory role from Celgene, Immunomedics, Genentech, Novartis, Pfizer, and Merck, institution-associated research funding from Merck, Pfizer, Tesaro, Genentech/Roche, G1 Therapeutics, and Daiichi Sankyo, and travel, accommodations, and expenses from Caris Life Sciences. AE, JX, and AH are employees of Caris Life Sciences. ZG reports stock/ownership in Caris Life Sciences. CI reports consulting/advisory role from Pfizer, Genentech/Roche, Novartis, AstraZeneca, Puma Biotechnology, Context Therapeutics, and Seattle Genetics, association with the speakers' bureau for Genentech, Pfizer, and AstraZeneca, received honoraria from Genentech/Roche, AstraZeneca, and Pfizer, and institution-associated research funding from Novartis, Pfizer, Genentech, and Tesaro. PP reports consulting/advisory role from Personalized Cancer Therapy, OncoPlex Diagnostics, Immunonet BioSciences, Pfizer, HERON, Puma Biotechnology, Sirtex Medical, Caris Life Sciences, and Juniper, association with the speakers' bureau for Genentech/Roche, patents: United States Patent Nos. 8,486,413, 8,501,417, 9,023,362, and 9,745,377, stock/ownership and leadership in Immunonet BioSciences, received honoraria from ASCO and Dava Oncology, and institution-associated research funding from Genentech/Roche, Fabre-Kramer, Advanced Cancer Therapeutics, Caris Centers of Excellence, Pfizer, Pieris Pharmaceuticals, and Cascadian Therapeutics. LS reports consulting/advisory role from Helsinn, Pfizer, Amgen, Genentech/Roche, BMS, Genomic Health, Myriad, AstraZeneca, Spectrum, Caris Life Sciences, and Lilly, association with the speakers' bureau for Coherus and PUMA, and has received research funding from Amgen, Spectrum, Genentech, Novartis, and AstraZeneca. MS reports association with the speakers' bureau for Astra Zeneca. WK reports stock/ownership interests and employment from Caris Life Sciences, consulting/advisory role from Merck. SS reports consulting/advisory role from Pieris Pharmaceuticals, Inivata, Tocagen, Genomic Health, Genentech/Roche, Lilly, Daiichi Sankyo, Molecular Templates, Athenex, and Silverback Therapeutics, travel, accommodations, and expenses from Inivata, Caris Centers of Excellence, Genentech/Roche, Lilly, Daiichi Sankyo, NanoString Technologies, Bristol-Myers Squibb, Novartis, and Caris Life Sciences, professional services agreement for IDMC from AstraZeneca, support for third party writing assistance from Roche, and institution-associated research funding from Genentech and Pfizer. FL reports consulting/advisory role from Bristol-Myers Squibb (unpaid), Jounce Therapeutics (unpaid), AstraZeneca (unpaid), and Pfizer/EMD Serono; received honoraria from ASCO; received travel, accommodations and expenses from Bristol-Myers Squibb and Genentech, has received research funding from Pfizer, Bristol-Myers Squibb, Jounce Therapeutics and Inivata, and institution-associated research funding from Calithera Biosciences, Immunomedics, Chugai Pharma USA, Regeneron, Inivata, and Tesaro. The remaining author declares that the research was conducted in the absence of any commercial or financial relationships that could be construed as a potential conflict of interest.
