# Supplementary material for: Prevalence of Phosphatidylinositol-3-Kinase (PI3K) Pathway Alterations and Co-alteration of Other Molecular Markers in Breast Cancer
Source: Front Oncol. 2020 Aug 31;10:1475. doi: 10.3389/fonc.2020.01475 (PMC7489343; doi:10.3389/fonc.2020.01475)
Supplement: Supplementary file 1 [file Data_Sheet_1.docx]

| **Table S1.** IHC antibody and threshold for positivity | | |
| --- | --- | --- |
| **Protein** | **IHC Positive Threshold** | **Antibody** |
| ER | ≥1+ and ≥1% | SP1 |
| PR | ≥1+ and ≥1% | 1E2 |
| HER2 | ≤1+ or =2+ and ≤10% or ≥3+ and >10% | 4B5 |
| PD-1 | ≥1 per high-powered field | MRQ-22 |
| PD-L1 LDT (Tumor cells) | ≥5% | SP142 |
| PD-L1 FDA CDx (Immune cells) | ≥1% | VENTANA PD-L1 (SP142) |
| MLH1 | ≥1+ and ≥1% | M1 |
| MSH1 | ≥1+ and ≥1% | G2191129 |
| MSH2 | ≥1+ and ≥1% | 44 |
| PMS2 | ≥1+ and ≥1% | EPR3947 |

| **Table S2.** Most common *PIK3CA-AKT1-PTEN* pathway alterations | | | | | | | | | | | |
| --- | --- | --- | --- | --- | --- | --- | --- | --- | --- | --- | --- |
| **Cohort** | | **All Subtypes** | | **HR+ Her2+** | | **HR- Her2+** | | **HR+ Her2-** | | **TNBC** | |
| **Total Samples, N** | | 4895 | | 263 | | 220 | | 2594 | | 1863 | |
| **Gene** | **Alteration** | **N** | **%** | **N** | **%** | **N** | **%** | **N** | **%** | **N** | **%** |
| ***PIK3CA*** | **Total On/Off label** | 1616 | 33.0% | 98 | 37.3% | 85 | 38.6% | 1075 | 41.4% | 358 | 19.2% |
|  | **Total On label** | 1204 | 24.6% | 81 | 30.8% | 61 | 27.7% | 782 | 30.1% | 280 | 15.0% |
|  | H1047R | 567 | 11.6% | 39 | 14.8% | 43 | 19.5% | 331 | 12.8% | 154 | 8.3% |
|  | E545K | 304 | 6.2% | 16 | 6.1% | 8 | 3.6% | 216 | 8.3% | 64 | 3.4% |
|  | E542K | 179 | 3.7% | 12 | 4.6% | 5 | 2.3% | 130 | 5.0% | 32 | 1.7% |
|  | H1047L | 70 | 1.4% | 4 | 1.5% | 0 | 0.0% | 49 | 1.9% | 17 | 0.9% |
|  | C420R | 28 | 0.6% | 4 | 1.5% | 0 | 0.0% | 19 | 0.7% | 5 | 0.3% |
|  | E545A | 16 | 0.3% | 1 | 0.4% | 2 | 0.9% | 11 | 0.4% | 2 | 0.1% |
|  | E545G | 15 | 0.3% | 0 | 0.0% | 1 | 0.5% | 11 | 0.4% | 3 | 0.2% |
|  | Q546R | 14 | 0.3% | 2 | 0.8% | 2 | 0.9% | 7 | 0.3% | 3 | 0.2% |
|  | E545D | 5 | 0.1% | 1 | 0.4% | 0 | 0.0% | 4 | 0.2% | 0 | 0.0% |
|  | H1047Y | 5 | 0.1% | 2 | 0.8% | 0 | 0.0% | 3 | 0.1% | 0 | 0.0% |
|  | Q546E | 1 | 0.0% | 0 | 0.0% | 0 | 0.0% | 1 | 0.0% | 0 | 0.0% |
|  | **Total Off label** | 412 | 8.4% | 17 | 6.5% | 24 | 10.9% | 293 | 11.3% | 78 | 4.2% |
|  | N345K | 74 | 1.5% | 2 | 0.8% | 3 | 1.4% | 61 | 2.4% | 8 | 0.4% |
|  | E726K | 45 | 0.9% | 0 | 0.0% | 2 | 0.9% | 36 | 1.4% | 7 | 0.4% |
|  | G1049R | 19 | 0.4% | 1 | 0.4% | 0 | 0.0% | 10 | 0.4% | 8 | 0.4% |
|  | E453K | 17 | 0.3% | 0 | 0.0% | 0 | 0.0% | 16 | 0.6% | 1 | 0.1% |
|  | G118D | 15 | 0.3% | 2 | 0.8% | 2 | 0.9% | 7 | 0.3% | 4 | 0.2% |
|  | Q546K | 15 | 0.3% | 0 | 0.0% | 0 | 0.0% | 13 | 0.5% | 2 | 0.1% |
|  | E110del | 11 | 0.2% | 0 | 0.0% | 0 | 0.0% | 6 | 0.2% | 5 | 0.3% |
|  | N1044K | 11 | 0.2% | 2 | 0.8% | 0 | 0.0% | 8 | 0.3% | 1 | 0.1% |
| ***AKT1*** | **Total** | 174 | 3.6% | 0 | 0.0% | 0 | 0.0% | 122 | 4.7% | 52 | 2.8% |
|  | E17K | 164 | 3.4% | 0 | 0.0% | 0 | 0.0% | 115 | 4.4% | 49 | 2.6% |
| ***PTEN*** | **Total** | 2897 | 59.2% | 9 | 3.4% | 4 | 1.8% | 1406 | 54.2% | 1478 | 79.3% |
|  | PTEN-IHC Negative | 2514 | 51.4% | 0 | 0.0% | 0 | 0.0% | 1212 | 46.7% | 1302 | 69.9% |
|  | T319fs | 37 | 0.8% | 2 | 0.8% | 1 | 0.5% | 16 | 0.6% | 18 | 1.0% |
|  | R130Q | 12 | 0.2% | 1 | 0.4% | 0 | 0.0% | 9 | 0.3% | 2 | 0.1% |

| **Table S3.** Patient and tumor characteristics by *PIK3CA*, *AKT1*, and *PTEN* mutation status | | | | | |
| --- | --- | --- | --- | --- | --- |
| **Variable** | ***PIK3CA*-MT** | ***AKT1*-MT** | ***PTEN*-MT** | ***PIK3CA-AKT1-PTEN*-MT** | ***PIK3CA-AKT1-PTEN-*WT** |
| All cases, N (%) | 1472 (30.1%) | 174 (3.6%) | 2684 (54.8%) | 3558 (72.7%) | 1337 (27.3%) |
| Female cases, N (%) | 1461 (99.3%) | 173 (99.4%) | 2661 (99.1%) | 3526 (99.1%) | 1319 (98.7%) |
| Male cases, N (%) | 11 (0.7%) | 1 (0.6%) | 23 (0.9%) | 32 (0.9%) | 18 (1.3%) |
| Median age, years (SD) | 60 (11.8) | 60 (11.8) | 57 (12.8) | 58 (12.7) | 57 (13.0) |
| Age range | 20-90 | 27-90 | 17-90 | 17-90 | 19-90 |
| Primary site, N (%) | 516 (35.1%) | 62 (35.6%) | 1071 (39.9%) | 1383 (38.9%) | 538 (40.2%) |
| Metastatic site, N (%) | 956 (64.9%) | 112 (64.4%) | 1613 (60.1%) | 2175 (61.1%) | 799 (59.8%) |
| Note: *PIK3CA*-MT, *AKT1*-MT, and *PTEN*-MT include any case with the specified mutation and do exclude double- or triple-mutant cases. *PIK3CA-AKT1-PTEN*-MT includes any case with a *PIK3CA*, *AKT1*, or *PTEN* mutation. *PIK3CA-AKT1-PTEN*-WT includes cases that are WT for *PIK3CA*, *AKT1*, and *PTEN*. | | | | | |

| **Table S4.** Co-Mutation Frequency with alpelisib On/Off-Label *PIK3CA* Alterations | | | | | | | | | | | | | |
| --- | --- | --- | --- | --- | --- | --- | --- | --- | --- | --- | --- | --- | --- |
| Pathway | Gene/ Protein | All subtypes (%) | | | | | | HR+HER2- (%) | | | | | |
|  |  | Alpelisib On | WT | Alpelisib Off | WT | Alpelisib On | Alpelisib Off | Alpelisib On | WT | Alpelisib Off | WT | Alpelisib On | Alpelisib Off |
| Homologous recombination | *BRCA1* | 0.3* | 4.0* | 0.8* | 4.0* | 0.3 | 0.8 | 0.5 | 1.5 | 0.6 | 1.5 | 0.5 | 0.6 |
|  | *BRCA2* | 3.4* | 5.1* | 3.2 | 5.1 | 3.4 | 3.2 | 3.4* | 6.5* | 3.1 | 6.5 | 3.4 | 3.1 |
|  | *PALB2* | 0.6 | 1.0 | 0.4 | 1.0 | 0.6 | 0.4 | 0.3* | 1.5* | 0.6 | 1.5 | 0.3 | 0.6 |
| DNA Damage Sensors | *CHEK2* | 2.5* | 1.3* | 5.3* | 1.3* | 2.5* | 5.3* | 3.1 | 1.9 | 7.5* | 1.9* | 3.1* | 7.5* |
| Possible predictors of CPI benefit | PD-1 | 36.2* | 53.9* | 45.0 | 53.9 | 36.2 | 45.0 | 34.9 | 41.2 | 33.3 | 41.2 | 34.9 | 33.3 |
|  | PD-L1 TC (SP142) | 4.9* | 6.7* | 6.2 | 6.7 | 4.9 | 6.2 | 1.6* | 4.0* | 2.6 | 4.0 | 1.6 | 2.6 |
|  | PD-L1 IC (SP142) | 14.4* | 34.2* | 7.1* | 34.2* | 14.4 | 7.1 | 8.6 | 18.6 | 5.0 | 18.6 | 8.6 | 5.0 |
|  | MSI | 0.4 | 0.7 | 1.5 | 0.7 | 0.4* | 1.5* | 0.5 | 0.6 | 1.7 | 0.6 | 0.5 | 1.7 |
|  | TMB-High (≥ 10/Mb) | 23.8* | 19.2* | 28.4* | 19.2* | 23.8 | 28.4 | 19.9 | 17.2 | 26.9* | 17.2* | 19.9* | 26.9* |
| Chromatin remodeling | *ARID1A* | 15.5 | 12.4 | 20.5* | 12.4* | 15.5 | 20.5 | 17.4 | 17.1 | 24.2 | 17.1 | 17.4 | 24.2 |
|  | *ARID2* | 1.0 | 0.6 | 0.8 | 0.6 | 15.5 | 20.5 | 1.1 | 0.5 | 1.1 | 0.5 | 1.1 | 1.1 |
| *RAS-RAF-MEK-ERK* | *HRAS* | 1.3* | 0.1* | 1.1* | 0.3* | 1.3 | 1.1 | 0.3 | 0.1 | 0.0 | 0.1 | 0.3 | 0.0 |
|  | *KRAS* | 2.8* | 1.3* | 1.1 | 1.3 | 2.8 | 1.1 | 2.6* | 1.2* | 1.7 | 1.2 | 2.6 | 1.7 |
|  | *NRAS* | 0.2 | 0.1 | 0.4 | 0.1 | 0.2 | 0.4 | 0.2 | 0.0 | 0.6* | 0.0* | 0.2 | 0.6 |
|  | *BRAF* | 0.5 | 0.4 | 1.1 | 0.4 | 0.5 | 1.1 | 0.3 | 0.5 | 1.1 | 0.5 | 0.3 | 1.1 |
| Others | *TP53* | 48.1* | 63.9* | 45.9* | 63.9* | 48.1 | 45.9 |  |  |  |  |  |  |
|  | *CDH1* | 15.6* | 5.7* | 18.0* | 5.7* | 15.6 | 18.0 | 19.4* | 9.2* | 21.4* | 9.2* | 19.4 | 21.4 |
|  | *NF1* | 7.6* | 3.8* | 7.7* | 3.8* | 7.6 | 7.7 |  |  |  |  |  |  |
|  | *PTEN* |  |  |  |  |  |  | 50.6 | 52.9 | 44.0* | 52.9* | 50.6 | 44.0 |
|  | *ERBB2* | 2.7 | 2.3 | 5.3* | 2.3* | 2.7* | 5.3* | 2.6 | 3.2 | 5.7 | 3.2 | 2.6* | 5.7* |
| Legend: On = mutation listed on alpelisib package insert.  Off= off-label pathogenic/ presumed pathogenic mutation.  WT= no pathogenic in *PIK3CA*.  TMB = tumor mutational burden.  *statistically significant difference between cohorts (p<0.05) | | | | | | | | | | | | | |
