# Supplementary material for: Prevalence of Phosphatidylinositol-3-Kinase (PI3K) Pathway Alterations and Co-alteration of Other Molecular Markers in Breast Cancer
Source: Front Oncol. 2020 Aug 31;10:1475. doi: 10.3389/fonc.2020.01475 (PMC7489343; doi:10.3389/fonc.2020.01475)
Supplement: Supplementary file 2 [file Data_Sheet_2.docx]

**Figure S1**

663

799

(54.3%)

1922

(71.6%)

94

70

(40.2%)

5

5

***PIK3CA*-MT**

**N=1472**

***AKT1*-MT**

**N=174**

***PTEN*-MT**

**N=2684**

**Figure S1.** *PIK3CA, AKT1, PTEN* alteration and co-alteration frequency
